# Integrating multiple immunogenetic data sources for feature extraction and mining somatic hypermutation patterns: the case of “towards analysis” in chronic lymphocytic leukaemia

**DOI:** 10.1186/s12859-016-1044-3

**Published:** 2016-06-06

**Authors:** Ioannis Kavakiotis, Aliki Xochelli, Andreas Agathangelidis, Grigorios Tsoumakas, Nicos Maglaveras, Kostas Stamatopoulos, Anastasia Hadzidimitriou, Ioannis Vlahavas, Ioanna Chouvarda

**Affiliations:** Department of Informatics, Aristotle University of Thessaloniki, Thessaloniki, Greece; Institute of Applied Biosciences, CERTH, Thessaloniki, Greece; Department of Immunology, Genetics and Pathology, Science for Life Laboratory, Uppsala University, Uppsala, Sweden; Division of Molecular Oncology and Department of Onco-Hematology, San Raffaele Scientific Institute, Milan, Italy; Lab of Computing and Medical Informatics, Medical School, Aristotle University of Thessaloniki, Thessaloniki, Greece

**Keywords:** Data integration, Feature extraction, List aggregation, Mutation patterns, somatic hypermutation, SHM, Chronic lymphocytic leukaemia, CLL

## Abstract

**Background:**

Somatic Hypermutation (SHM) refers to the introduction of mutations within rearranged V(D)J genes, a process that increases the diversity of Immunoglobulins (IGs). The analysis of SHM has offered critical insight into the physiology and pathology of B cells, leading to strong prognostication markers for clinical outcome in chronic lymphocytic leukaemia (CLL), the most frequent adult B-cell malignancy. In this paper we present a methodology for integrating multiple immunogenetic and clinocobiological data sources in order to extract features and create high quality datasets for SHM analysis in IG receptors of CLL patients. This dataset is used as the basis for a higher level integration procedure, inspired form social choice theory. This is applied in the *Towards Analysis*, our attempt to investigate the potential ontogenetic transformation of genes belonging to specific stereotyped CLL subsets towards other genes or gene families, through SHM.

**Results:**

The data integration process, followed by feature extraction, resulted in the generation of a dataset containing information about mutations occurring through SHM. The *Towards analysis* performed on the integrated dataset applying voting techniques, revealed the distinct behaviour of subset #201 compared to other subsets, as regards SHM related movements among gene clans, both in allele-conserved and non-conserved gene areas. With respect to movement between genes, a high percentage movement towards pseudo genes was found in all CLL subsets.

**Conclusions:**

This data integration and feature extraction process can set the basis for exploratory analysis or a fully automated computational data mining approach on many as yet unanswered, clinically relevant biological questions.

## Background

Immunity is the capability of the human organism to defend from the attack of environmental agents that are foreign to itself and are potentially harmful. Those foreign elements could be viruses, bacteria and various other substances [1]. Immunity can be divided in innate and acquired.

The term innate immunity refers to all those parts of the human body that serve as the first line of defense. It is always present and available in healthy individuals and its main aim is to avoid the entry of foreign invaders [2]. Some of its components are the skin, the mucous membranes and the cough reflex. Its most important features are speed (within hours), non-specificity, lack of memory and limited effectiveness.

On the other hand, acquired immunity serves as the second line of defense and is activated if a foreign invader (substance) manages to surpass the first line. The initial contact with the foreign substance triggers the immune response, which leads to the activation of lymphocytes (a type of white blood cells) and their products, such as antibodies, which are the main elements of the acquired immunity. After the initial immunization, the individual is capable to resist a subsequent attack from the same invader, which is called antigen. Acquired immunity is characterized by slow response time, memory and antigen-specificity [1].

B lymphocytes or B cells are one of the two main cell types of the acquired immune system (the other being T lymphocytes). The main function of B cells is specific antigen recognition, antibody production and immune response activation in order to eliminate danger and maintain the homeostasis of the host [3]. In each cell, lying at the heart of this process is a unique B-cell receptor (BcR), a multimeric complex, which is mainly characterized by its immunoglobulin (IG) molecule [3].

Each IG molecule is composed of two identical heavy chains (HCs) and two identical light chains (LCs), each subdivided into two regions with different functionality, namely the variable (V) and constant (C) domain: in more detail, the V domain is responsible for antigen binding, while the C domain has an effector function through the determination of the IG isotype. Each V domain is comprised of 7 areas of variable diversity. Of those, the areas with relatively limited diversity are known as the framework regions (FRs), whereas the highly variable areas are known as the complementarity determining regions (CDRs) and confer each IG molecule a unique specificity [4].

The V domain of the IG HC and LC of each B cell is generated by a random process of DNA rearrangement known as V(D)J recombination [5–7] which brings together one each of distinct variable (V), diversity (D; for HCs only) and joining (J) genes, leading to a great variety of combinations. It has been estimated that the combinatorial events of the IG heavy (*IGH)*, IG kappa (*IGK*) and IG lambda (*IGL*) gene loci create greater than 1.6 × 10^6^ possible combinations for BcR IGs (http://www.imgt.org/IMGTrepertoire). A second set of diversification is also induced following antigen selection with somatic hypermutation (SHM) and class switch recombination (CSR), both occurring within secondary lymphoid organs [8, 9]. SHM and CSR have been estimated to increase the potential for diversity up to 10^12^ different IGs, each with a distinct primary sequence and, likely, antigen specificity [3].

The term SHM refers to the introduction of mutations within rearranged V(D)J genes at a rate of at least 10^6^ – fold higher than the spontaneous rate of mutagenesis elsewhere in the genome. Most mutations are single nucleotide substitutions rather than deletions or insertions and occur at an estimated rate of 1 per 1000 base pair per generation [10].

The analysis of SHM has offered critical insight into the physiology and pathology of B cells. Focusing on malignancies of mature B cells, particular imprints of SHM are widely accepted as evidence for antigen encounter while ongoing SHM leading to intraclonal diversification of IG genes is supporting the concept of continued interactions with antigen throughout the natural history of the clone i.e., also post-transformation [11]. From a clinical perspective, the study of SHM has been established as one of the strongest prognostic markers for clinical outcome [12, 13] in chronic lymphocytic leukemia (CLL), the most frequent adult B-cell malignancy. It is now well established that the mutational status of the rearranged IGHV genes directly correlates with patient survival, with unmutated IGHV genes relating to more aggressive clinical course and shorter survival than mutated IGHV genes. Of note, immunogenetic studies of CLL have also revealed a remarkably biased IGHV gene repertoire as well as a differential impact of SHM depending on IGHV gene usage with IGHV3-7 and IGHV4-34 carrying a high mutational load in contrast to IGHV1-69 which exhibits very few mutations [14]. Moreover, unrelated CLL cases were found to carry remarkably similar VH CDR3 sequences and also sharing recurrent SHM, thus further corroborating the concept of antigen selection in CLL ontogeny [15–17]. Based on the existence of common motifs, CLL patients can be assigned to different “stereotyped subsets” with distinct clinicobiological profiles [18, 19].

The actual mechanisms of SHM bias have been studied from different viewpoints, including the regions where they most frequently occur (framework-FR or complementarity determining regions -CDR) [20], the codons involved and the physicochemical properties implicated [21], as well as the difference among stereotyped subsets [14], especially focusing on those expressing the IGHV3-21 and IGHV4-34 genes. Other methods [22] address modeling of SHM and assess the degree to which such models explain variance of real cases.

In this work, SHM is studied from a different perspective. Based on data driven modeling, minimizing a priori assumptions about the process and regarding each IGHV gene as a whole, we focus on detecting the potential direction of transformation of these genes, and investigate similarities or differences in clinically or biologically relevant groups. The implemented methodology consists of the following main steps. At first, different immunogenetic and clinicobiological data are integrated in order to extract features and create high quality datasets for somatic hypermutation (SHM) analysis in the clonotypic immunoglobulin (IG) receptors of CLL patients. Next, a data integration method is proposed, following the principles of social voting, under the concept that all patient samples are ‘equivalent’ experiments to be taken into account. The integrated group points at the preferential directions of transformation. The virtue of the proposed approach is illustrated via the case of stereotyped subset – specific “Towards Analysis”, which is our attempt to detect patterns of mutation-based transformation of genes towards other genes or gene families through SHM. The choice to focus on subsets was made on the grounds that these represent homogeneous groups, thus helping to overcome the incapacitating heterogeneity of CLL, and, also, because of postulated differences in their ontogeny from non-stereotyped fraction of CLL [17].

This data mining approach can extend to different directions and can set the basis for an in-depth investigation of a series of as yet unanswered clinically relevant biological questions, which could be of great value in translational medicine, given the great prognostic value of SHM in CLL.

## Methods

This section is organized as follows. Initially, we present the three integrated data sources. Then, we describe in detail the data pre-processing step that aims to integrate the different data sources and ensure data quality. Finally, we explain the feature extraction process and the generated datasets and we conclude this section by an in-depth description of all steps of the *Towards Analysis*, from data preparation to the final step of Borda aggregation, the latter being a special case of data integration where the integrated data are ranked or scored lists of elements. The study was conducted in accordance with the Declaration of Helsinki and approved by CERTH Institutional Review Board on 18/08/2014.

### Integrated data sources

#### IMGT/HighV-QUEST output

The first source of data is a collection of results obtained from IMGT/HighV-QUEST tool output analysis [23] in a single run of a set of sequences. IMGT/V-QUEST is a highly customized and integrated system for the standardized analysis of the immunoglobulin (IG) and T cell receptor (TR) rearranged nucleotide sequences. The tool output consists of different files providing information such as functionality, V, D and J genes identified after alignment with the reference directory (germline genes), percentage identity of the identified genes with the germline (GI%), the positioning of nucleotide substitutions and the possible amino acid changes that they may induce, information about amino acid properties and the nucleotide and amino acid gapped, i.e., aligned, sequences [23, 24].

#### Reference dataset

**Fig. 1:**
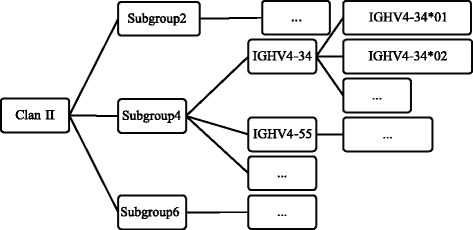
Reference dataset. Reference dataset is organized in a hierarchical manner of alleles-genes-subgroups-clans. Figure presents the sub-tree specific for IGHV4-34*01 and IGHV4-34*02 alleles

The reference dataset consists of the amino acid and nucleotide germline sequences of *Homo sapiens* IGHV genes obtained from IMGT/GENE-DB [25]. These are organized in a hierarchical manner of alleles-genes-subgroups-clans (Fig. 1). A gene can have more than one allele. For instance, the IGHV4-34 gene has thirteen alleles (e.g., IGHV4-34*01, IGHV4-34*02 etc.). The number after the letter “V” in the IMGT nomenclature, denotes the subgroup that this allele belongs to. There are seven subgroups named from one to seven (IGHV1, IGHV2,…IGHV7). A clan is a set of subgroups. There are three clans for human IGHV genes. Clan I: *Homo sapiens* IGHV1, IGHV5 and IGHV7 subgroup genes; clan II: *Homo sapiens* IGHV2, IGHV4 and IGHV6 subgroup genes; clan III: *Homo sapiens* IGHV3 subgroup.

#### Classification of patient sequences to stereotyped subsets

**Fig. 2:**
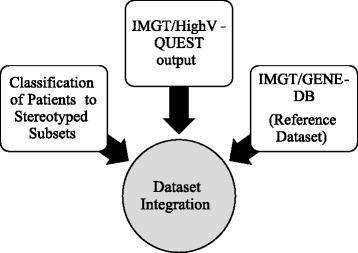
Integrated data sources

The third data source is data from the clinicobiological database that holds various types of clinical and biological patient data, including the assignment of patients to subsets expressing identical clonotypic B Cell Receptors [18, 19]. The latter is an example of contextual information that can distinguish groups of patients with regards to their unique biological features and clinical behavior. It can also be helpful for data mining depending on the question under investigation. A graphic display of data integration is shown in Fig. 2.

### Data preprocessing

**Fig. 3:**
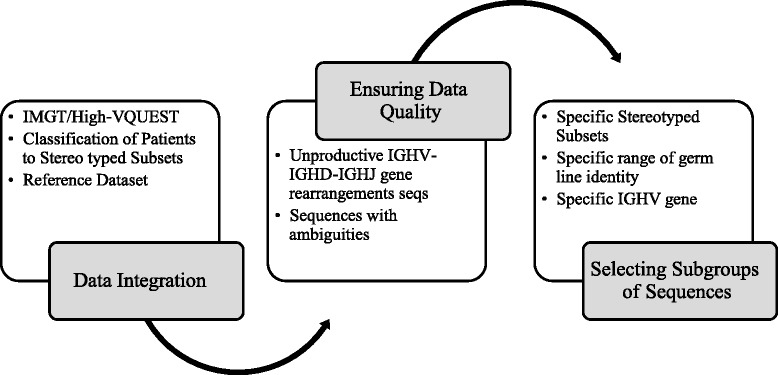
Data preprocessing step. This step leads from raw data to selected data for feature extraction

The first step in the feature extraction process is the data preprocessing step (depicted in Fig. 3) whose aim is twofold: first, to integrate the different data sources and second, to ensure the highest data quality.

#### Data integration

The analysis is patient-orientated and, therefore, the key behind the data integration is the patient unique ID in the patient related data sources. The first step of data integration is the parsing of the IMGT/HighV-QUEST output files and the clinicobiological dataset. Information obtained for each patient sequence includes: Patient unique ID, functionality of the IGHV-IGHD-IGHJ gene rearrangement (productive/unproductive), closest germline V-GENE and allele, germline identity (GI%), the nucleotide and amino acid gapped sequence according to IMGT numbering [26], and the list of nucleotide mutations and amino acid changes.

#### Filtering integrated data

In this step, several filters have been developed in order to ensure high data quality and choose the appropriate subsets/subgroups for further analysis. More specifically, *Data Quality Filters* analyze and subsequently exclude unqualified patient sequences such as those with sequence ambiguities or unproductive IGHV-IGHD-IGHJ gene rearrangement sequences. Then, *Subgroup Selection Filters* direct the analysis to a specific subgroup of the analyzed sequences. The latter may concern the selection of sequences that belong to specific stereotyped subsets, have specific range of IGHV gene germline percentage identity, or carry the same IGHV gene. In addition, analysis can be focused on specific VH domain subregions (e.g., heavy variable CDR1).

### Identification of somatic hypermutations shared with another germline gene

Based on the assumption that a nucleotide substitution (mutation) may show a trend from one germline to another and that a particular clonotypic rearranged IGHV sequence may actually represent the intermediate step between the two germlines, we herein refer to a mutation as shared with another germline gene (in short *SH*) if the nucleotide introduced by SHM is also present at the germline in question at the exact same position. Moreover, in this analysis we refer to the closest V-GENE and allele germline originally identified by the IMGT/HighV-QUEST output as “sequence before the mutation (*sBm*)” and to the patient sequence as “sequence after the mutation (*sAm*)”. We call the germline with the aforementioned SHs “Towards Germline” (*TowG*) indicating the potential movement (increase of similarity) from the *sBm* sequence towards this germline. Finally, we define as “*non-SH*” a mutation that resulted in a nucleotide that cannot be found in any germline sequence at this particular position.

The core and most important part of the analysis is the identification of SHs. In this part of the analysis, all patient sequences (*sAm*) are compared with all the germlines from the reference directory dataset in order to classify mutations as *SH* or *non-SH*. It is important to mention that for the purposes of the present study the term *SH* makes sense only with regards to a germline. Hence, a mutation cannot be defined as *SH* without referring to a corresponding *TowG*.

### Feature extraction and dataset generation

The previously described *SH* analysis, resulted in two mutation based sets, one for *SH* mutations and one for *non-SH* mutations, both sharing the same structure: (sequence ID, mutation, Towards Germline). These sets serve as the baseline for feature extraction and more specifically, for the construction process which will result to three different mutation-based datasets.

The first dataset is called *“SH Position Dataset (SHPD)”* and contains 34 features. Each entry of the *SHPD* is a *SH* with a *TowG*. The features that have been constructed or included from the integration phase for each entry are as follows: Patient Unique ID; patient assignment to a stereotyped subset; *sBm*, germline identity; *TowG*; number of *SH* mutations with the *TowG*; mutational position number; the number of the codon that the *SH* mutation belongs to; VΗ domain region information (i.e., FR1, CDR1, etc.); information whether the mutation occurred in a hotspot motif, if it was a transition or transversion and, finally, whether it was replacement or silent. Furthermore, features were constructed, based on the IMGT scientific chart [27], describing all properties of the amino acids encoded by the triplet in which the mutational position belonged in all three steps under investigation, i.e., *sBm*, *sAm* and *TowG*. Included properties were hydropathy, volume, chemical, physicochemical and charge.

The second generated dataset called “*Non-SH Position Dataset (nonSHPD)*” differs from *SHPD* in including information about the functionality subgroup where the new property (i.e., the property of the amino acid in the *sAm*) can be found. The previously described datasets were constructed in order to study in detail the mutations (shared and non-shared) individually.

**Fig. 4:**
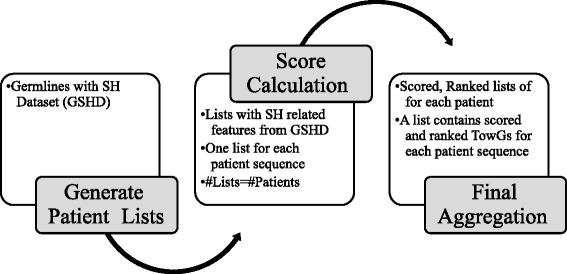
Towards analysis steps. This process leads from the dataset generated based on feature extraction process to the final aggregated results

The third dataset called “*Germlines with SH Dataset (GSHD)*” contains 8 features. Each entry of the dataset expresses a couple *sBm* and *TowG*, while the remaining features include information related to these two values. More specifically, the features are: *sBm*, *sAm*, identity of *sBm* and *sAm*, *TowG*, identity of *TowG* and *sBm* and the number of *SH* mutations. The features of this dataset have been specifically selected in order to study the potential transformation of the *sBm* sequences to *TowGs*. This is what we call *Towards Analysis* and is described in detail in the following paragraphs and is depicted in Fig. 4.

### Towards analysis

#### Voting systems

Our approach is inspired by social choice and voting theory. A voting system is a method by which voters make a choice between different available options. The study of formally defined voting systems is called social choice theory or voting theory and it is a subfield combining political sciences, economics and mathematics. Until now, many voting systems have been proposed and can be roughly distinguished by the method voters express their preferences. For instance, in the most common system, which is called first-past-the-post or simple plurality, voters select only one option. In this study, we are interested in preferential voting systems or ranked voting systems in which voters rank options in a hierarchy on the ordinal scale. One of the most important topics in this voting system is the problem of how votes are counted and aggregated to yield a final result. This is what we call *the ranking aggregation problem*. An analogous definition has been suggested by Dwork et al. [28] who define ranking aggregation as a problem of computing a “consensus” ranking of the alternatives, given the individual ranking preferences of several voters.

The ranking aggregation problem can be intuitively divided into two categories based on the length and the number of the lists to be aggregated [29]. The first category, which also appeared first, is the problem of aggregating many short lists, as in the case of elections. More recently, the problem of aggregating a few long lists gained more popularity due to the importance of its fields of application. The first field is the World Wide Web and the task is the aggregation of results obtained by different search engines [28]. The second is the field of bioinformatics and the analysis of high throughput, omics-scale, biological data. More specifically, rank aggregation methods have gained popularity in combining results that occur from different gene expression experiments [30–35]. The use of ranking aggregation methods to combine results from different studies can be called “high level” meta-analysis because the researcher does not have to deal with the underling raw data but only with the ranked lists obtained from those experiments [29].

In this study, we approach the *Towards Analysis* as a ranking aggregation problem, i.e., the aggregation of few long lists. By analogy, in our analysis each patient sequence is a voter. Each voter scores i.e., ranks, a set of choices, which are the *TowGs*. The scoring of the *TowGs* is based on the *SH* mutation analysis described previously. The Towards Analysis problem is to aggregate these “votes” in order to obtain a final consensus ranking. The approach is explained in detail in the following paragraphs.

*Towards analysis* is based on results obtained from the feature extraction process and more specifically based on the dataset *Germlines with SH Dataset (GSHD)* described above. Our approach intends to formulate the *Towards Analysis* problem as a ranking aggregation problem, by: a) constructing a number of different towards lists, each one corresponding to a different patient sequence (voter), b) aggregating them in order to obtain the consensus lists of the alternatives and c) identifying patterns of potential transformation or movement of *IGHV* gene germlines towards other *IGHV* gene germlines through SHM.

#### Constructing raw data lists

First, we split the initial dataset in many short lists. The number of the resulting lists is equal to the number of patient sequences in the *GSHD* and each list corresponds to one patient sequence (*sAm*). From the available features in *GSHD* we select only those features which indicate the towards *IGHV* germline gene, the number of *SH* mutations and the number of mutations in this patient sequence (*M*). The list *Li* of patient *i* is described by metadata information that includes features from *GSHD* (Patient Unique ID, *sBm* and patient stereotyped subset), mainly for directing the analysis to specific subgroups of sequences, and the number of identified mutations *Mi*. The element *j* of *Li*, *Lij*, is *Lij = {SHij, Towards Allele_j}*. The list *Li* represents one vote for the final aggregation of votes per *sBm*, i.e., per aligned IGHV allele of the sequence, e.g., IGHV4-34*01.

#### Constructing lists for aggregation

The purpose of this step is to transform the initial raw lists *Li* to lists in their final form which is suitable for aggregation, and specifically, a towards list *LGi* of genes (not alleles) accompanied with a score. For patient i the k^th^ element is *LGik = {score_ik, Gene_k}.*

In this step, the algorithm transforms each ranked list of all towards *IGHV* germline genes based on a score. The alleles are replaced by their corresponding gene and the mutation related information (*SH* mutation and *M*) is replaced by a score. In order to avoid biased results occurring from IGHV genes with many alleles, we calculated the number of *SH* mutations of each gene *SH*_*ik*_ as the arithmetic mean of the *SH* mutations of its alleles, i.e., the expected *SH* mutations among the members of this set.

The scoring system is based on the SHs, the concepts of “available movement” and “initial movement capability” and finally a “selectivity” factor and is given by the following formula:

1 $$ scor{e}_{ik}=\frac{S{H}_{ik}\ast S{H}_{ik}}{M_i\ast maxS{H}_k\ast selS{c}_k} $$

The formula can be seen as a product of three factors. Firstly, given that M_i_ is the number of mutations in a patient sequence and represents the maximum “available movement” in a particular patient case, the first factor (SH_ik_/M_i_) represents the portion of the “available movement” achieved. Secondly, given that maxSH_k_ is the sequence dissimilarity between the *sBm* and *TowG* and expresses the “initial movement capability” between *sBm* and *TowG*, the second factor (SH_ik_/maxSH_k_) is the portion of the “initial movement capability” achieved. Finally, selSc_k_ is the number of *TowG* genes that are also found to have the same number of *SH* with this gene and express the selectivity among genes.

After the score calculation of each *TowG*, each *LGi* list of patient sequence *i* which corresponds to a *sBm* germline sequence, contains a list of scored Towards Genes (the pairs *score_ik, gene_k*) expressing the potential movement of the *sBm* towards these genes.

#### List preprocessing

##### *Filtering*

In this step, filters have been developed to direct the analysis to a specific subgroup of patient sequences. This is performed via the selection from the initial pool of lists of those belonging to a specific subset and/or to specific IGHV Gene.

##### *Homogenizing*

After the filtering step, a homogenization step takes place. This step is essential because different lists could contain different towards germline genes and consequently the list aggregation could not be performed. In homogenizing step, all lists are transformed to contain the union genes found in all the selected for aggregation lists. After the transformation, the newly introduced genes in each list are scored with zero value.

##### *Normalizing LG*_*i*_

As stated below, in our analysis we considered each patient sequence as a separate experiment and more importantly equivalent with all other patient sequences independently of the number of mutations introduced by SHM. To achieve this, we normalized each list score by scaling to [0, 1]. The normalized score *s*_*i*_ of the *TowG* (i) is given by the following formula:

2 $$ Normalized\left({S}_i\right)=\frac{s,-{S}_{min}}{S_{max}\mathit{\hbox{-}}{S}_{min}} $$

After the normalization, the first towards germline with score 1 indicates the maximum movement that the *sBm* of the corresponding patient sequence can achieve. The normalization of scores results in the normalized lists *LGN*_*i*_.

#### Borda inspired aggregation

The final aggregation of the lists *LGN*_*i*_ can be made using various Borda-inspired methods [29]. In our approach we used a variant of the original method proposed by Borda [36] which is the most intuitive. Although in most cases, each element’s score (Borda score of each element) is the simple rank of the element in each list, in situations where other additional information is available (in our case the previously calculated score), Borda score can be redefined in order to represent the additional information in the aggregation process [29]. In our approach, the final score of each element in the final rank is the arithmetic mean of each element score across the aggregated normalized lists *LGN*_*i*_. The aggregation function for the score of each element, s_i_, in the n lists is the following:

3 $$ f\left({S}_1,\kern0.5em {S}_2,\dots, \kern0.5em {S}_n\right)=\frac{1}{n}{\displaystyle {\sum}_i^n{S}_i} $$

## Results and Discussion

### Data sources description

Distinctive SHM patterns amongst CLL cases have been previously reported, especially regarding subsets with stereotyped BCRs [14]. With this analysis we sought for differentiation trends concerning shared mutations amongst CLL patients expressing IGHV4-34 clonotypic B cell receptors, namely subsets #4, #11, #16, #29 and #201 [18].

The integrated dataset was based on the following 3 datasets: Firstly, the alignment results obtained from IMGT/HighV-QUEST output for a set of 20331 CLL cases; secondly 341 reference germline genes obtained by IMGT/GENEBD Version 3.1.0 (4 April 2014) and finally the clinicobiological information for the 20331 cases.

In order to ensure data quality we discarded through filtering all unproductive rearrangement sequences, sequences that contain ambiguities and incomplete CDR1 and upstream regions. Then, we selected only sequences that were classified to the above mentioned stereotyped subsets (#4 – 164 cases; #11 – 16 cases; #16 – 44 cases; #29 – 43 cases; and #201 – 45 cases).

Concerning the reference dataset, we discarded all orphans and those alleles that were partial in 5’. The resulted dataset contained 284 reference germline alleles (234 Functional; 38 pseudo genes; and 12 open reading frames).

### Analysis results and discussion

The towards analysis was performed separately for each subset, in order to investigate not only the occurring differences but also the similarities between the subsets under investigation, potentially alluding to similar selective pressures. For each subset, the analysis resulted in a scored list of *TowGs*. The score of each *TowG* indicates the overall movement of the IGHV4-34 gene belonging to a specific subset. Towards analysis was performed in the whole IGHV gene region, and was repeated in the conserved gene area, i.e., in positions where all alleles of the specific gene have the same nucleotide. The rationale was to investigate whether towards mutations affect the conserved regions, and can thus be regarded as a gene-biased phenomenon, related to the germline codon composition, or rather present some allele specific properties.

**Fig. 5:**
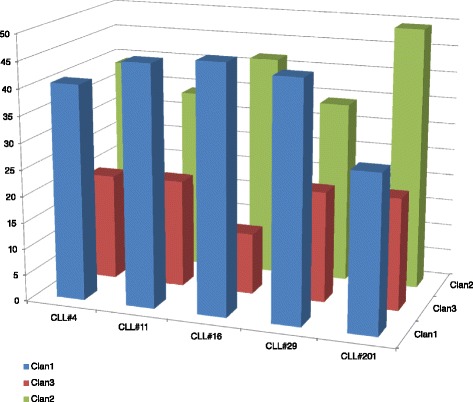
Expected movement per clan (whole gene area)

**Fig. 6:**
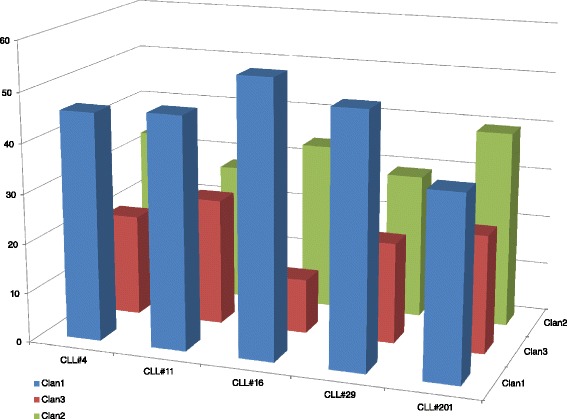
Expected movement per clan (conserved gene area)

**Fig. 7:**
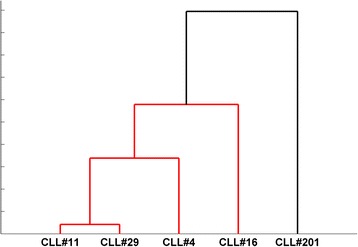
Clustering of subsets based on the distances of movement per clan (whole gene area)

**Fig. 8:**
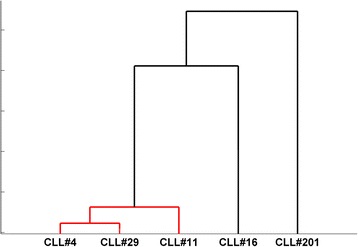
Clustering of subsets based on the distances of movement per clan (conserved gene area)

Firstly, we tried to investigate the subset movement towards one of the three different IGHV gene clans. To calculate this, we summarized all scores of the *TowGs* belonging to a specific clan, as the average score of that clan’s genes, i.e., the expected movement per clan for every subset. The results are shown in Table 1 for the whole gene area and Table 2 for the conserved area. The differences between subsets per clan are shown in Figs. 5 and 6. Figures 7 and 8 present the clustering of subsets based on the distances of movement per clan via a dendrogram.

**Table 1:** Expected movement per clan (Whole gene area)

	CLL#4	CLL#11	CLL#16	CLL#29	CLL#201
Clan1	40.64	45.42	46.52	44.82	29.47
Clan3	20.08	20.35	11.55	20.94	21.18
Clan2	39.27	34.23	41.93	34.24	49.36

**Table 2:** Expected movement per clan (Conserved gene area)

	CLL#4	CLL#11	CLL#16	CLL#29	CLL#201
Clan1	45.86	46.70	55.15	50.51	36.71
Clan3	20.43	25.51	10.92	20.25	23.69
Clan2	33.71	27.78	33.93	29.23	39.60

In both cases, the distinct behaviour of subset #201 is apparent. The main difference between the two dendrograms is the interchange of subset #4 and subset #11 as the one closest to subset #29. In the whole IGHV region analysis subset #29 was closer to subset #11. In non-conserved analysis this is no longer the case, suggesting that subset #11 and subset #29 have many similarities in non-conserved positions. Moreover, it is obvious that the analysis in the conserved region produced a more solid cluster of subsets #4, #11 and #29 differentiating them from subsets #16 and #201.

**Fig. 9:**
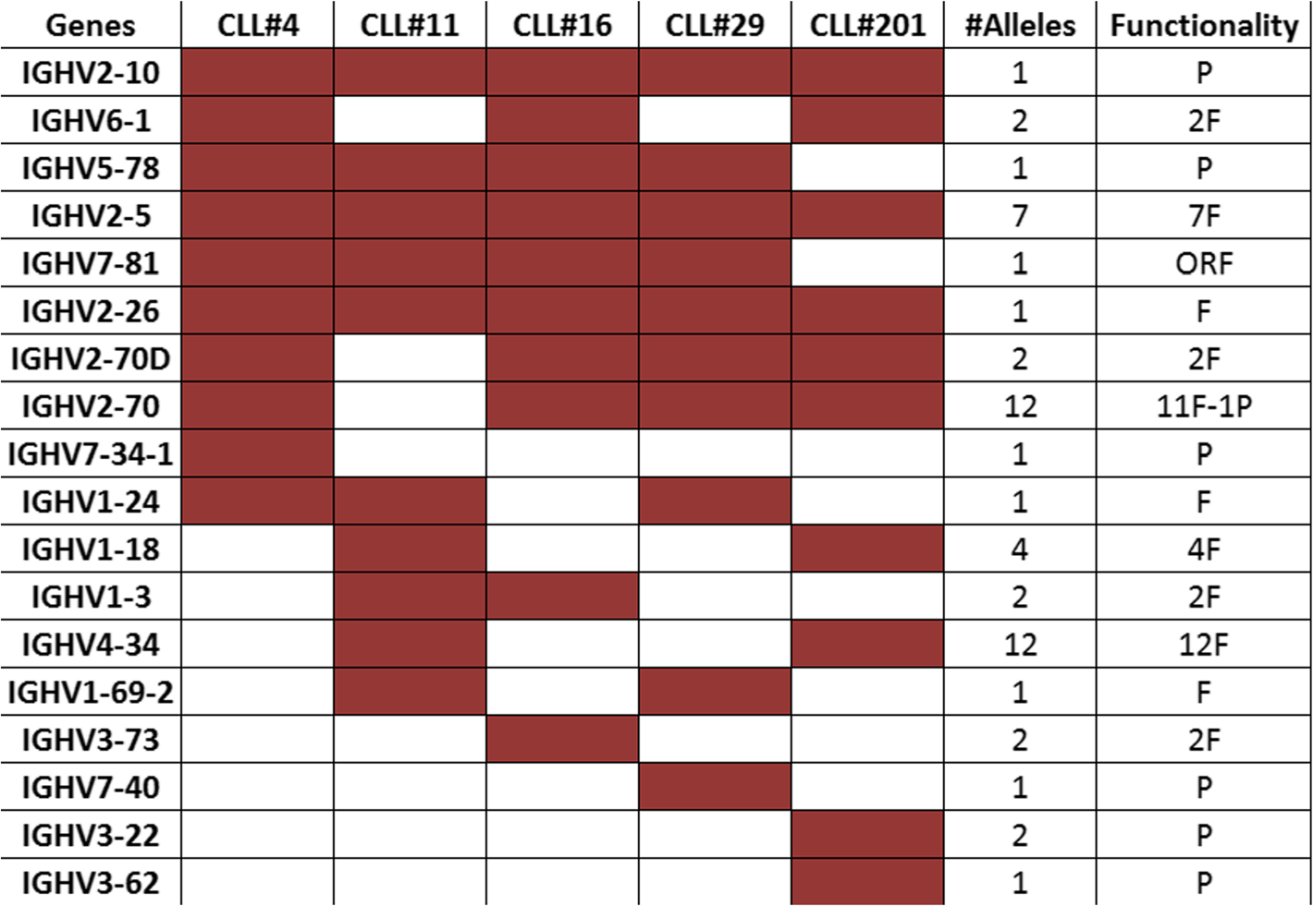
First ten toward germlines (TowGs) for every subset (whole gene area). The set containing the first ten *TowGs* for every subset. It is important to mention that this graph does not express a ranked list, but rather a union of the highly ranked genes across subsets (with potentially different ranking per subset). The red color indicates that the gene in this row can be found in the top ten of the corresponding subset in the column. For that reason, every column has exactly ten cells (whole gene area)

**Fig. 10:**
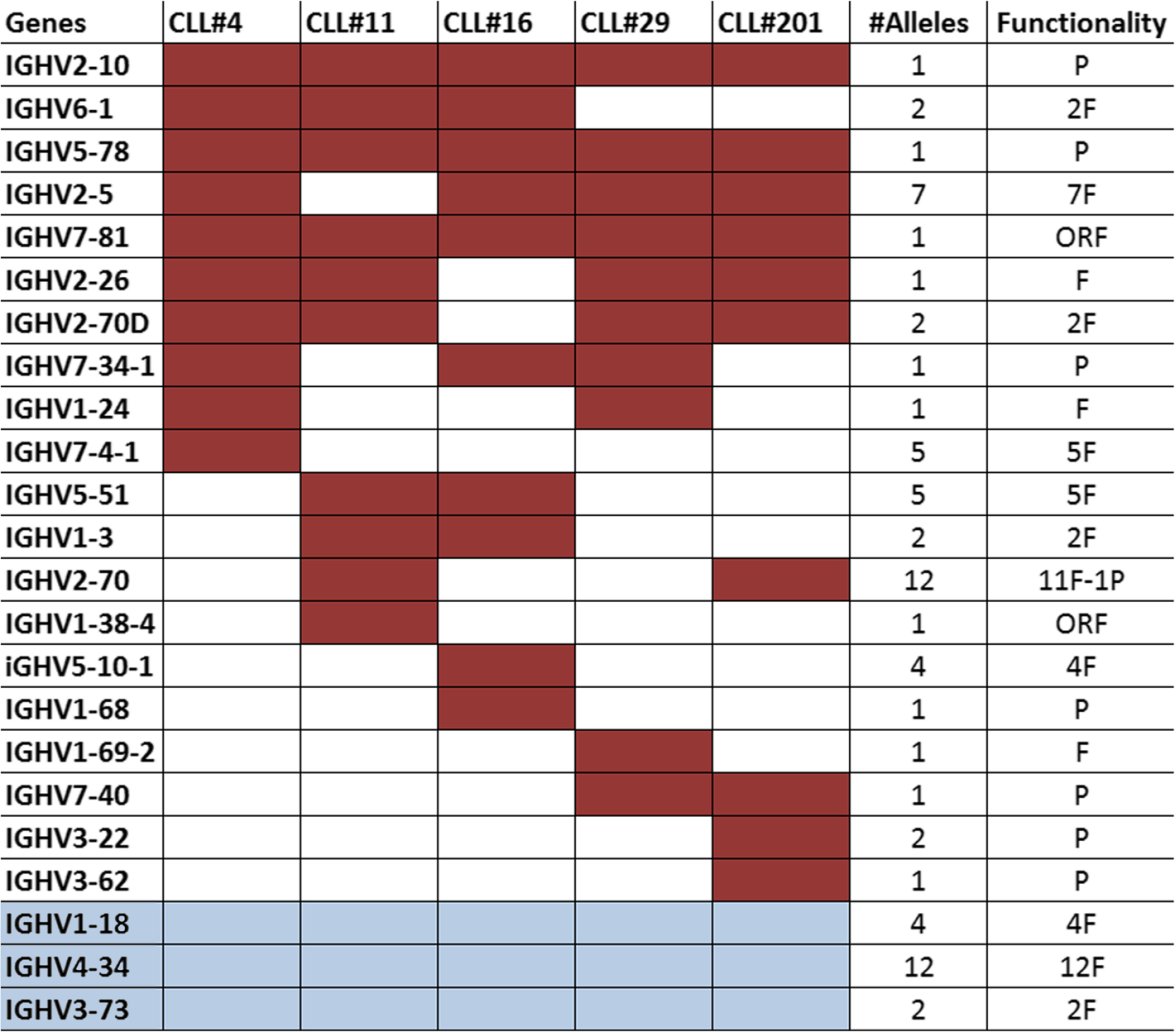
First ten toward germlines (TowGs) for every subset (conserved gene area). The set containing the first ten *TowGs* for every subset. It is important to mention that this graph does not express a ranked list, but rather a union of the highly ranked genes across subsets (with potentially different ranking per subset). The red color indicates that the gene in this row can be found in the top ten of the corresponding subset in the column. For that reason, every column has exactly ten cells. Blue cells denote difference from the whole gene analysis, i.e., genes that are not in top10 in conserved analysis (conserved area)

The second question was the investigation of movement towards individual genes. In order to have a more complete perspective of each *TowG* movement, we considered the sum of all scores in a list as the whole movement of this subset and thus we normalized each score to the total sum of scores, expressing the percentage movement per gene. In Figs. 9 and 10 we present the set containing the first ten *TowGs* for every subset with the number of alleles of every *TowG* and their functionality.

From the graph it becomes clear that there are many common highly ranked *TowGs* among subsets. Firstly, IGHV2-10, IGHV2-5 and IGHV2-26, all belonging to Clan II (same as IGHV4-34), can be found in the top - 10 of every subset. Moreover, IGHV5-78 and IGHV7-81 genes are in the top-10 list in four out of the five subsets (missing from subset#201) and that also goes for IGHV2-70 and IGHV2-70D genes (missing from subset#11). Another interesting observation is that the presence of pseudogenes in this graph is extremely high given the very low number of pseudogenes compared to the number of functional ones.

Comparing the above-mentioned figure (Fig. 9) with the one produced from the conserved analysis (Fig. 10) we can see only minor differences. The high presence of pseudogenes is also obvious here. The small changes in the figure pattern could suggest different mutation patterns in the non-conserved area. For instance gene IGHV2-5 is present in all subsets in the analysis of the whole gene region, but it is absent in the allele-conserved area for subset#11, suggesting that maybe there are important mutation patterns in the non-conserved area for this subset.

**Fig. 11:**
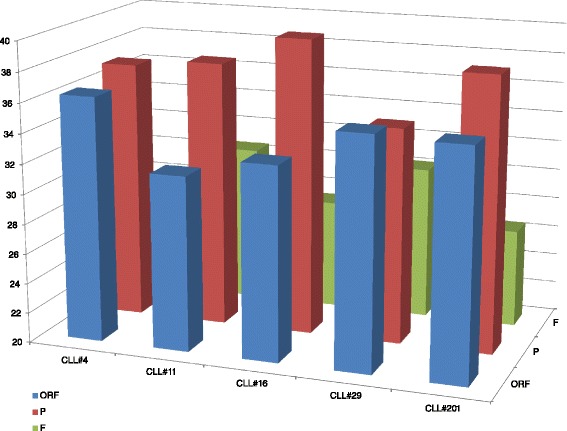
Expected movement per functionality (whole gene area)

The observation about the high presence of pseudogenes prompted us to investigate the percentage movement per functionality. Given that several genes include both functional and non-functional alleles (pseudogenes), it was impossible to generalize that a gene has a specific functionality. For this reason we performed the same analysis, but instead of generalizing to the gene, we investigated the movement per allele, and finally aggregated per functionality. The results are presented in Table 3 and Fig. 11. To account for the higher number of functional alleles and make those movements comparable, we calculated the average expected movement per functionality. Interestingly, we see that for all subsets the average expected movement is higher in pseudogenes. While a concrete interpretation would need more extended studies, it is worth noting that pseudogenes have been found to play a role as DNA templates in other mechanisms for genetic diversification, like gene conversion and class switch recombination [37]. The relevance of this observation in a highly complex and specialized system such as a mature B cell undergoing specialized genomic reorganization changes after antigen encounter remains to be fully elucidated.

**Table 3:** Expected movement per functionality (Whole gene area)

	CLL#4	CLL#11	CLL#16	CLL#29	CLL#201
ORF	36.33	31.66	32.91	35.37	35.18
P	37.28	37.76	39.75	34.39	38.29
F	26.39	30.57	27.33	30.24	26.53

## Conclusion

In this paper we present a methodology for integrating multiple immunogenetic and clinocobiological data sources in order to extract features and create high quality datasets for SHM analysis in the clonotypic immunoglobulin receptors of CLL patients. This can set the basis for exploratory analysis or a fully automated computational data mining approach on many as yet unanswered, clinically relevant biological questions, considering that SHM is one of the most robust prognostic indicators in CLL. We also introduce the *Towards analysis*, which is our attempt to investigate the potential “ontogenetic transformation” of genes belonging to specific stereotyped subsets towards other genes or gene subgroups and clans, through SHM.

The methodological innovation of this work is mainly the integration of the three closely related immunogenetic data sources for the generation of a rich SHM-related dataset, with the final aim of data analysis and knowledge extraction. Moreover, we firstly, to our knowledge, used the ranking aggregation approach and the formalization of voting systems, (sequences voting for gene mutation preferences), to give insights into the problem of the potential ontogenetic “gene transformation”. In order to achieve this we proposed a score which is the quantification of the gene movement through *SH*.

Our future work concerns a more thorough investigation of the presented results, mainly towards the investigation of the differences between the *SH* and *non-SH* (e.g., with respect to regional CDR/FR or in amino acid properties), and the differences between mutations leading to pseudogenes or functional genes. This also involves the feature extraction and the generation of new datasets, tackling our raised questions at the amino acid level. This work is considered as the basis for further investigation of SHM-related biological questions in the broader field of immune processes in health and disease.

## Abbreviations

BCR: B – cell receptor
CDR: complementarity determining regions
CLL: chronic lymphocytic leukemia
FR: Framework region
GSHD: germlines with shared mutations dataset
IG: immunoglobulin
IMGT: the international immunogenetics information system
non-SH: a mutation that resulted in a nucleotide that cannot be found in any germline sequence at this particular position
nonSHPD: non-shared mutations position dataset
sAm: sequence after mutation
sBm: sequence before the mutation
SH: a mutation is referred as shared with another germline gene if the nucleotide introduced by SHM is also present at the germline in question at the exact same position
SHM: somatic hypermutation
SHPD: shared mutations position dataset
TowG: towards germline
